# Vismodegib in Locally Advanced Basal Cell Carcinoma in Slovenia

**DOI:** 10.1159/000525612

**Published:** 2022-07-27

**Authors:** Tanja Mesti, Maša Sever, Janja Ocvirk

**Affiliations:** ^a^Department for Solid Tumors, Institute of Oncology Ljubljana, Ljubljana, Slovenia; ^b^Medical Faculty, University of Ljubljana, Ljubljana, Slovenia

**Keywords:** Basal cell carcinoma, Gorlin Goltz syndrome, Vismodegib, Hedgehog pathway inhibitors, Treatment-emergent adverse events, Multidisciplinary approach, Early supportive care

## Abstract

**Background:**

Vismodegib is a first-in-class inhibitor of the hedgehog pathway for treatment of locally advanced basal cell carcinoma (laBCC) and metastatic BCC.

**Objectives:**

The purpose of this study is to report outcomes of patients with laBCC, with basal cell carcinoma nevus syndrome (Gorlin Goltz syndrome [G-G Syn]) treated with vismodegib in routine clinical practice in Slovenia in 8.3-year period.

**Methods:**

In this retrospective cohort study, we analyzed baseline characteristics, outcomes, and treatment-related adverse events from locally advanced BCC. The patients were divided into two cohorts: 39 laBCC or multiple BCC patients and 7 patients with G-G Syn who were treated with vismodegib from November 2012 till January 2021.

**Results:**

During 100-month period, 46 patients were diagnosed with laBCC (26), multiple BCC (13), and G-G Syn (7), all inappropriate for surgery or radiotherapy. Baseline characteristics: median age was 72.8 years in laBCC + multiple BCC cohort and 47.4 years in G-G Syn cohort. The objective response rate was 80% in laBCC + multiple BCC and 86% in G-G Syn cohort. Disease control rate (DCR) was 95% in laBCC + multiple BCC and 100% in G-G Syn cohort. Median duration of treatment was 9.9 months (range: 1.5–43.1) in laBCC and multiple BCC cohort and 19.5 months (range: 3.6–94.1) in G-G Syn cohort. Majority of treatment-emergent adverse events (TEAEs) in laBCC or multiple BCC cohort were grade 1 or 2 (96%), only 4% of AEs were grade 3. Majority of TEAEs in G-G Syn cohort were also grade 1 or 2 (87%), 13% of AEs were grade 3. No grade 4 or 5 vismodegib-related AEs were reported.

**Conclusion:**

Vismodegib has shown meaningful efficacy with DCR from 95% to 100% in patients with laBCC, multiple BCC, and G-G Syn in Slovenia. TEAEs were successfully alleviated with multidisciplinary approach and early supportive care.

## Introduction

Basal cell carcinoma (BCC) is the most common human malignancy representing ∼80% of all nonmelanoma skin cancers [1]. Although characterized by local spreading and a low tendency to metastasize and successfully treated by surgery or, less frequently, radiation therapy, if left untreated, it may progress to advanced BCC (aBCC) including locally advanced (laBCC) and metastatic BCC (mBCC). Nevoid BCC syndrome, also known as Gorlin Goltz syndrome (G-G Syn), is a hereditary condition characterized by multiple BCC and other abnormalities. BCCs in this syndrome behave in the same manner as sporadic BCCs and rarely metastasize. In laBCC, where tumor invades and destroys local tissue, surgery and radiation may be inappropriate due to significant loss of vital function, intolerable morbidity, and disfigurement [2]. Vismodegib, a small molecule hedgehog pathway inhibitor, is indicated for the treatment of mBCC or laBCC in which the disease has recurred after surgery or in which patients are not candidates for surgery or radiation therapy [3]. Although data from pivotal ERIVANCE trial with vismodegib and study assessing the use of vismodegib in a large cohort of patients representative of routine clinical practice (the SafeTy Events in VIsmodEgib study − STEVIE) show that vismodegib is a safe and effective treatment option for patients with laBCC and mBCC, additional information about real-world use of this drug is still needed [4, 5, 6]. With this retrospective cohort study, we aimed to review management of patients with laBCC, including patients with nevoid BCC syndrome, also known as G-G Syn treated at our department with vismodegib focusing on management of side effects, assessment of outcomes, and comparison of our findings with the literature.

## Materials and Methods

The study was approved by the Institutional Ethics Committee (ERIDNPVO-0006/2020). We performed a retrospective analysis of the clinical characteristics, vismodegib treatment patterns, and adverse events in 46 patients with laBCC, multiple BCC, and nevoid BCC syndrome − G-G Syn, treated at the Oncology institute between November 2012 and January 2021. Patients with G-G Syn were analyzed separately from laBCC + multiple BCC. There were no organ transplant recipients or patients with mBCC included in this study. Population of 46 patients represents all vismodegib-treated patients in Slovenia since vismodegib EMA approval with the exception of 1 pediatric patient treated at the Pediatric clinic Ljubljana.

Vismodegib treatment was considered in patients for which curative resection was deemed unlikely and/or when significant morbidity and/or deformity were anticipated with surgery. All of the patients received oral vismodegib 150 mg per day until the disease progressed or they developed unacceptable toxicity. Treatment interruptions and discontinuations as well as adverse events were recorded at regular hospital visits. Common Terminology Criteria for Adverse Events v5.0 were used to grade AEs.

The therapeutic effect was monitored by measuring the total diameter of the tumors, the diameter of the biggest lesion, and the number of lesions according to medical documentation and regularly taken clinical photographs. Vismodegib treatment efficacy was assessed by objective response rate (ORR), disease control rate (DCR), and duration of vismodegib treatment (DoT). The ORR was defined as sum of complete response (CR) and partial response (PR). DCR was defined as sum of CR, PR, and stable disease. DoT was defined as the length of time from the start of vismodegib treatment, till the end of the vismodegib treatment (treatment failure or unacceptable treatment toxicity). The median DoT was assessed.

## Results

### Baseline Characteristics

**Fig. 1:**
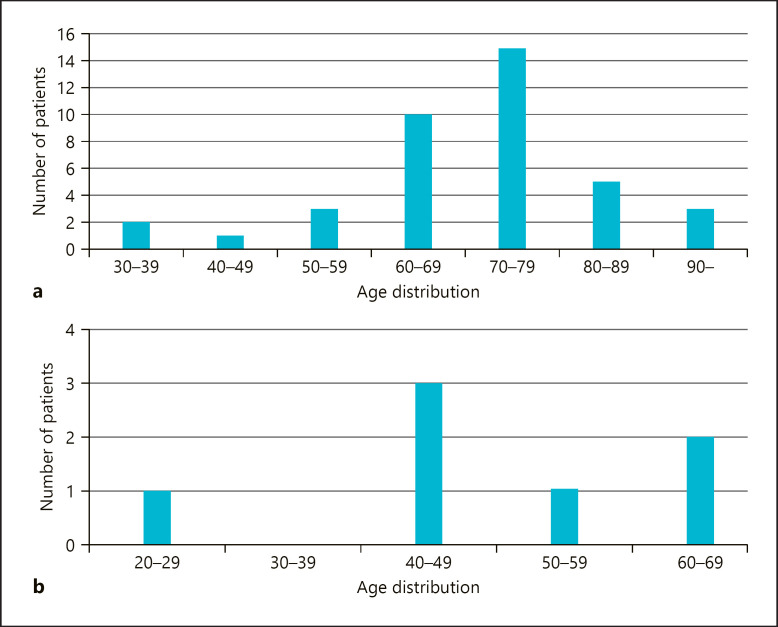
Patient age distribution at the beginning of vismodegib treatment for laBCC and multiple BCC cohort (**a**) and G-G Syn cohort (**b**).

We treated 46 patients with clinically and histologically confirmed laBCC (26), multiple BCC (13), or G-G Syn (7). Median age was 72.8 years (range: 31.1–97.5) in laBCC and multiple BCC cohort and 47.4 years (range: 21.9–61.3) in G-G Syn cohort. Patient age distribution varied among cohorts (shown in Fig. 1).

**Fig. 2:**
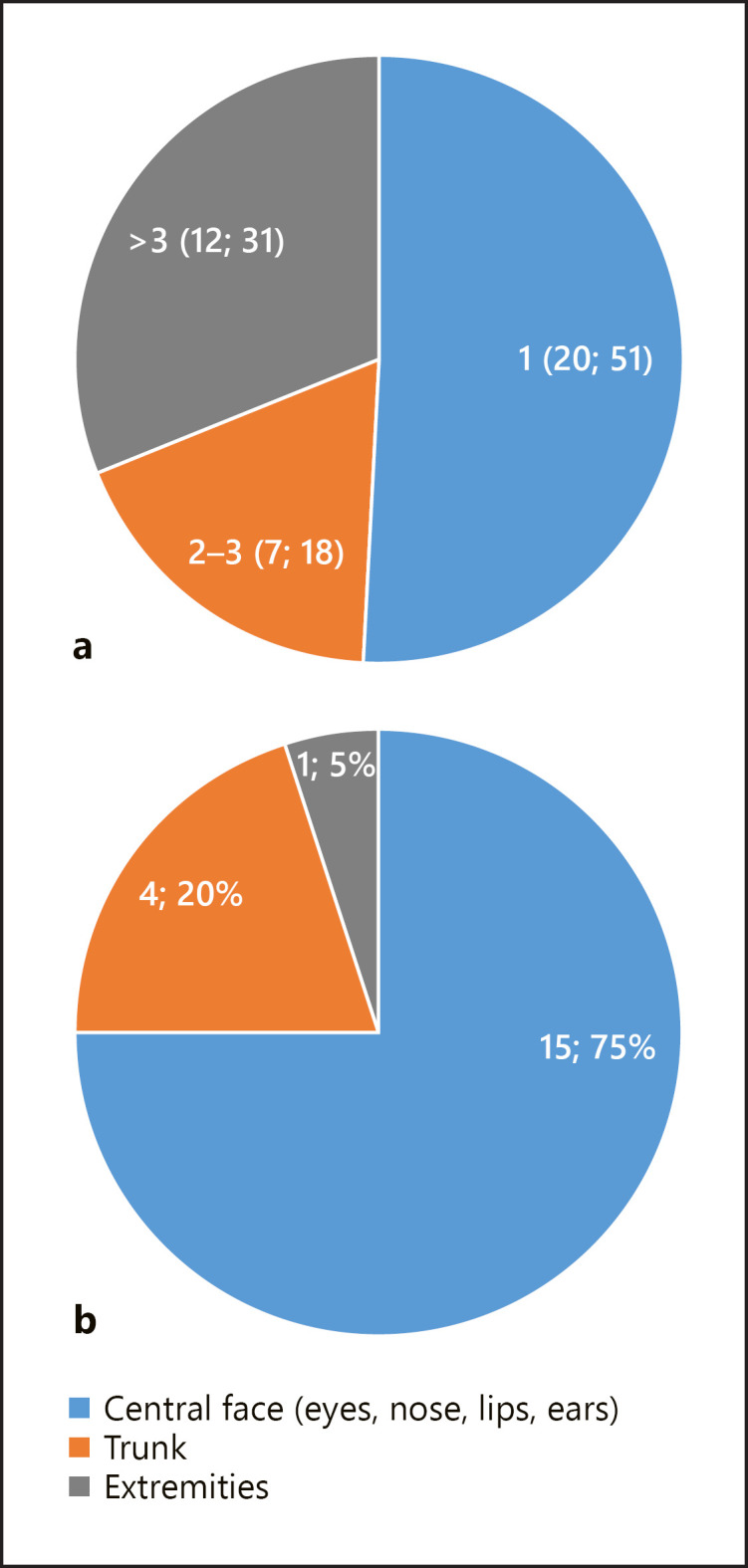
Number of target lesions (*n*, %) (**a**) and localization of target lesion in case of one lesion (*n*, %) (**b**) in patients with laBCC and multiple BCC. laBCC, locally advanced BCC.

Fifty-six percent of patients in laBCC and multiple BCC cohort were females; majority (64.1%) of patients were previously treated by surgery and/or radiotherapy; 51.3% of patients had 1 lesion with predominant localization in central face (eyes, nose, lips, or ears in 75% of patients), 18% had 2–3 lesions, and 31% more than 3 lesions (shown in Fig. 2). One patient was previously treated for malignant melanoma, colorectal cancer, and squamous cell carcinoma (SCC). Fifty-seven percent of patients in G-G Syn cohort were males; all patients were previously treated by surgery and/or radiotherapy.

### Vismodegib Efficacy

#### Patients with laBCC and Multiple BCC

The ORR was 80%, CR was 18%, and PR was 62% in laBCC and multiple BCC cohort. DCR for this cohort was 95%. Median DoT was 9.9 months (range: 1.5–43.1).

#### Patients with G-G Syn

**Fig. 3:**
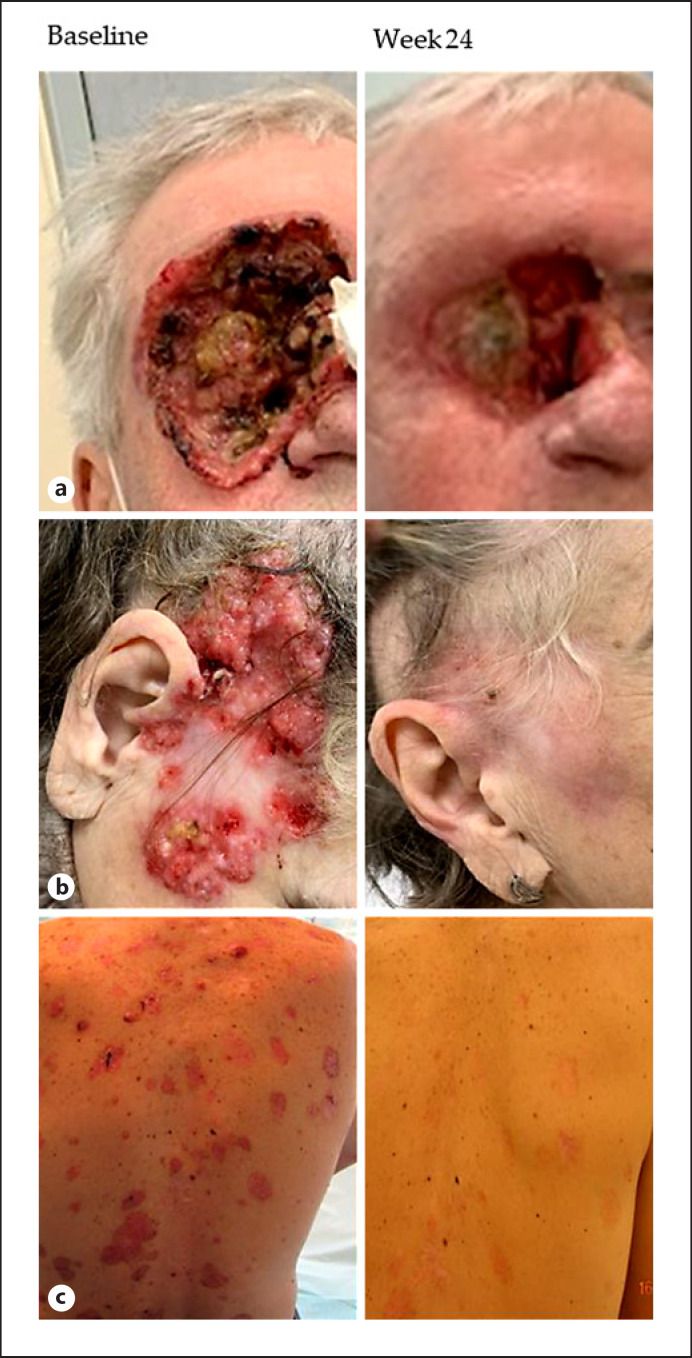
Photographs of patients treated with vismodegib with laBCC (**a**), multiple BCC (**b**), and G-G Syn (**c**).

In patients with G-G Syn, ORR was 86%, while CR and PR were 14% and 72%, respectively. DCR was 100%, and DoT was 19.5 months (range: 3.6–94.1). Visible reductions in tumor size and improvement in appearance were present for the majority of patients in both the cohorts (shown in Fig. 3).

### Safety

At the time of analysis in laBCC or multiple BCC cohort of patients, treatment had been interrupted due to treatment-emergent adverse events (TEAEs) in 9 (23.1%) patients, 12 (30.7%) patients were still on treatment. Reasons for permanent discontinuation of vismodegib treatment in 23 patients were CR in 7 (30%), TEAEs in 7 (30%), cancer in 4 (17%), disease progression in 2 (9%), for 2 patients information was missing, and 1 patient died.

At the time of analysis in G-G Syn cohort, treatment had been interrupted in 50% of patients for management of toxicity and 50% of patients were still on treatment. Adverse events of any grade were reported in 79.5% of patients in laBCC or multiple BCC cohort and 71.4% in G-G Syn cohort.

Majority of TEAEs in laBCC or multiple BCC cohort were grade 1 or 2 (96%), 4% of TEAEs were grade 3: muscle cramps in 3 patients; respiratory infection, vomiting, and anemia in 1 patient each. Majority of TEAEs in G-G Syn cohort were also grade 1 or 2 (87%), 13% of TEAEs were grade 3: muscle cramps in 2 patients, decreased weight and diarrhea in 1 patient each. No grade 4 or 5 vismodegib-related TEAEs were reported.

Serious adverse events (SAEs) not related to the vismodegib treatment were reported in 7 out of 46 patients (15.2%): two cases of SCC and one case of angiosarcoma, melanoma, cholangiocarcinoma, and intracerebral hemorrhage each. In the first SCC case, histological change to basosquamous cell carcinoma was observed, and while primary tumor minimally increased in size, metastatic SCC node occurred. In the second SCC case, patient developed simultaneously cholangiocarcinoma. One death case was registered due to fall and multiple fractures, followed by sepsis and multiorgan failure. The TEAEs in laBCC or multiple BCC and G-G Syn cohort are presented in Tables 1 and 2.

## Discussion

Vismodegib was the first approved targeted therapy in the treatment of mBCC or laBCC in patients who are not candidates for surgery or radiotherapy. We present our single-center retrospective cohort study of vismodegib for the treatment of laBCC and G-G Syn in 46 Slovenian patients during 8.3-year span.

In the pivotal ERIVANCE phase-II, single-arm, 2-cohort, multicenter clinical trial, with aim to evaluate the efficacy and safety of vismodegib in patients with aBCC, the efficacy analysis in 63 histologically confirmed patients with laBCC the ORR was 60.3%, while in the MIKIE randomized, regimen-controlled, double-blind, phase 2 trial, with aim to compare the efficacy and safety of two different regiments of vismodegib and which enrolled patients with multiple BCC, including those with G-G Syn (*n* = 42), ORR ranged from 54% to 62% [5, 7, 8]. In the safety STEVIE single-arm, multicenter, open-label study, with 1,232 patients enrolled, ORR was 68.5% in laBCC and 81.7% in patients with G-G Syn [6]. Efficacy in our analysis was closest to the data of the STEVIE study (ORR in laBCC/multiple BCC and G-G Syn was 80% and 86%, respectively). This similarity is probably because the STEVIE patient population reflected more real-world setting, as patients were elderly (median age for laBCC in the STEVIE study and our cohort were 72.0 and 72.8 years, respectively) and response rate was investigator-assessed. The difference in response rate between our study cohorts and aforementioned trials could be due to small size of the cohorts but also due to an early and therefore successful supportive care. Our data are similar to the results of other small-size cohorts treated with vismodegib in everyday practice (ORR 86%, *n* = 22) [9]. In addition, due to small size of the cohorts, the analysis according to the histological subtypes was not performed.

The DoT was 9.9 months in laBCC and multiple BCC group, and this is comparable to DoT of 8.6 months in the STEVIE study [6]. Interruption of treatment rate in this cohort was 24%, similar to the one reported in ERIVANCE trial, in which 21.2% of patients had to interrupt treatment [5]. DoT of 19.5 months in our G-G Syn cohort was also similar to the data from the STEVIE study. There was interruption of treatment (50%) due to TEAEs. The G-G Syn subgroup analysis indicates that patients with Gorlin syndrome responded better to treatment, with a considerably higher DoT of 19.5 months than patients without Gorlin syndrome (9.9. months), which might be a result of these patients being younger and having a better ECOG performance status than patients without Gorlin syndrome.

The most frequent TEAEs observed in at least 10% of patients were dysgeusia, muscle cramps, alopecia, decreased weight, and decreased appetite, demonstrating a consistent safety profile with that previously reported for vismodegib. The majority of TEAEs were grade 1 or 2, occurring early in the course of treatment. In a small cohort with G-G Syn, long-term exposure to vismodegib (up to 86.4 months) was associated with worsening severity (13% of TEAEs were grade 3), but no worsening in the frequency of TEAEs. We reported SAEs in 15.2% of patients, which is lower but still comparable to SAE rate of 23.2% in laBCC patients in the STEVIE study. The incidence of cutaneous SCC was 4.3% in our study and 4.2% in the study STEVIE, which is consistent with the meta-analysis estimating the risk for subsequent SCC in patients with a history of BCC that reported estimated proportion of 4.3% [10].

The most common reason for our patients to temporarily or permanently discontinue treatment with vismodegib was the appearance of TEAEs. TEAEs were treated early and successfully with multidisciplinary approach and use of pharmacologic and nonpharmacologic supportive care. The multidisciplinary approach at our institution incorporates multidisciplinary teams of nutritionists, physical medicine and rehabilitation specialists, dermatologists, neurologists, psychotherapists, and clinical pharmacologists. Besides treatment interruption, management of toxicity included spasmolytics (tizanidine), nerve stabilizers, magnesium supplements and exercise, cooling and heating to alleviate muscle spasms. For taste disturbance, decreased appetite, and weight loss, the nutritional consultation and support was provided and nutritional supplements, megestrol acetate, and corticosteroids were periodically prescribed. Most instances of muscle spasm resolved 1–3 months after treatment, and most occurrences of ageusia, dysgeusia, and alopecia resolved by 6–12 months after treatment. The possibility of metatypical changes in histology suggests careful evaluation of primary tumor including the biopsy of areas resistant to treatment.

## Conclusion

With this retrospective cohort study of patients treated with vismodegib in Slovenia for locally advanced basal cell carcinoma in a 100-month period (8.3 years), we add additional real-world data to this topic. In 46 patients treated with laBCC, with multiple BCC or G-G Syn, we have achieved very good efficacy with ORR from 80 to 86%, DCR from 95 to 100%, and DoT from 9.9 to 19.5 months (laBCC and multiple BCC and G-G Syn, respectively). Most of the treated patients experienced TAES grade 1–2 (96% and 87% for laBCC and multiple BCC, and G-G Syn cohort, respectively), with grade 3 ranging from 4% for laBCC and multiple BCC cohort to 13% for G-G Syn cohort. TEAEss were treated early and successfully with multidisciplinary approach and use of pharmacologic and nonpharmacologic supportive care.

Therefore, close follow-up of patients during treatment is necessary to manage TEAEs, assure compliance, and regularly assess continued benefit of treatment. A multidisciplinary approach is essential not only when making a decision to treat patients with aBCC with vismodegib but also in the successful management of toxicity.

## Key Message

A multidisciplinary management of toxicity in patients with aBCC with vismodegib prolongs survival.

## Statement of Ethics

This study protocol was reviewed and approved by the Institutional Ethics Committee, approval number (ERIDNPVO-0006/2020). For this retrospective study, the written informed consent was not required. The written consent was provided from the patients for which images were used in this publication. This study protocol was reviewed and approved by the Institutional Ethics Committee, approval number (ERIDNPVO-0006/2020). For this retrospective study, the written informed consent was not required.

## Conflict of Interest Statement

The authors have no conflicts of interest to declare.

## Funding Sources

The study was supported by the Slovenian Research Agency (ARRS), P3-0321.

## Author Contributions

All authors Tanja Mesti, Maša Sever, and Janja Ocvirk contributed equally to conceptualization; data gathering, modeling, and analysis; interpretation of the data; and drafting, writing, and editing of this paper.

## Data Availability Statement

The research data for this study are not publicly available on legal and ethical grounds − Regulation (EU) 2016/679 − General Data Protection Regulation (GDPR) protection of natural persons with regard to the processing of personal data and the free movement of such data. Further inquiries can be directed to the corresponding author.

## Figures and Tables

**Table 1:** Most common TEAEs in laBCC or multiple BCC cohort (reported in ≥10% of patients), *N* = 39

TEAE	Grade 1, *N* (%)	Grade 2, *N* (%)	Grade 3, *N* (%)	Grade 4, *N* (%)	Proportion of patients with TEAE, *N* (%)
Dysguesia	13 (33.3)	15 (38.5)	0 (0)	0 (0)	28 (71.8)
Muscle cramps	9 (23.1)	12 (30.8)	3 (7.7)	0 (0)	24 (61.5)
Alopecia	21 (53.8)	3 (7.7)	0 (0)	0 (0)	24 (61.5)
Decreased weight	8 (20.5)	8 (20.5)	0 (0)	0 (0)	16 (41.0)
Decreased appetite	5 (12.8)	8 (20.5)	0 (0)	0 (0)	13 (33.3)
Elevated AST and ALT <1.5× ULN	4 (10.3)	1 (2.6)	0 (0)	0 (0)	5 (12.8)
Fatigue	4 (10.3)	1 (2.6)	0 (0)	0 (0)	5 (12.8)

AST, aspartate aminotransferase; ALT, alanine aminotransferase; ULN, upper limit of normal.

**Table 2:** All reported TEAEs in G-G Syn cohort (reported in ≥10% of patients), *N* = 7

TEAE	Grade 1, *N* (%)	Grade 2, *N* (%)	Grade 3, *N* (%)	Grade 4, *N* (%)	Proportion of patients with TEAE, *N* (%)
Muscle cramps	1 (14.3)	1 (14.3)	2 (28.6)	0 (0)	4 (57.1)
Alopecia	1 (14.3)	3 (42.9)	0 (0)	0 (0)	4 (57.1)
Diarrhea	1 (14.3)	1 (14.3)	1 (14.3)	0 (0)	3 (42.9)
Dysguesia	0 (0)	3 (42.9)	0 (0)	0 (0)	3 (42.9)
Decreased weight	1 (14.3)	0 (0)	1 (14.3)	0 (0)	2 (28.6)
Abdominal spasms	1 (14.3)	1 (14.3)	0 (0)	0 (0)	2 (28.6)
Dry skin	2 (28.6)	0 (0)	0 (0)	0 (0)	2 (28.6)
Arthralgia	0 (0)	1 (14.3)	0 (0)	0 (0)	1 (14.3)
Insomnia	0 (0)	1 (14.3)	0 (0)	0 (0)	1 (14.3)
Nausea	0 (0)	1 (14.3)	0 (0)	0 (0)	1 (14.3)
Recurrent conjunctivitis	0 (0)	1 (14.3)	0 (0)	0 (0)	1 (14.3)
Recurrent respiratory infections	0 (0)	1 (14.3)	0 (0)	0 (0)	1 (14.3)
Recurrent sinusitis	0 (0)	1 (14.3)	0 (0)	0 (0)	1 (14.3)
Recurrent subcutaneous abscesses	0 (0)	1 (14.3)	0 (0)	0 (0)	1 (14.3)
Fatigue	1 (14.3)	0 (0)	0 (0)	0 (0)	1 (14.3)
Flatulence	1 (14.3)	0 (0)	0 (0)	0 (0)	1 (14.3)
Decreased appetite	1 (14.3)	0 (0)	0 (0)	0 (0)	1 (14.3)
